# Aspartate tightens the anchoring of staphylococcal lipoproteins to the cytoplasmic membrane

**DOI:** 10.1002/mbo3.525

**Published:** 2017-09-13

**Authors:** Nimerta Kumari, Friedrich Götz, Minh‐Thu Nguyen

**Affiliations:** ^1^ Interfaculty Institute of Microbiology and Infection Medicine (IMIT) Microbial Genetics University of Tübingen Tübingen Germany; ^2^ Institute of Microbiology University of Sindh Jamshoro Pakistan; ^3^ School of Biological and Food Technology Hanoi University of Science and Technology Hanoi Vietnam

**Keywords:** Aspartate position +2, gram‐positive bacteria, lipoprotein, lipoprotein release, Staphylococcus

## Abstract

In gram‐negative bacteria, the ABC transporter LolCDE complex translocates outer membrane‐specific lipoproteins (Lpp) from the inner membrane to the outer membrane. Lpp possessing aspartate (Asp) at position +2 are not translocated because it functions as a LolCDE avoidance signal. In gram‐positive bacteria, lacking an outer membrane and the Lol system, Lpp are only anchored at the outer leaflet of the cytoplasmic membrane. However, the release of Lpp particularly in pathogenic or commensal species is crucial for immune modulation. Here, we provide evidence that in *Staphylococcus aureus* Asp at position +2 plays a role in withholding Lpp to the cytoplasmic membrane. Screening of published exoproteomic data of *S. aureus* revealed that Lpp mainly with Gly or Ser at position +2 were found in exoproteome, but there was no Lpp with Asp+2. The occurrence of Lpp with Asp+2 is infrequent in gram‐positive bacteria. In *S. aureus* USA300 only seven of the 67 Lpp possess Asp+2; among them five Lpp represented Lpl lipoproteins involved in host cell invasion. Our study demonstrated that replacing the Asp+2 present in Lpl8 with a Ser enhances its release into the supernatant. However, there is no different release of Asp+2 and Ser+2 in *mpr*F mutant that lacks the positive charge of lysyl‐phosphatidylglycerol (Lys‐PG). Moreover, substitution of Ser+2 by Asp in SitC (MntC) did not lead to a decreased release indicating that in staphylococci positions +3 and +4 might also be important for a tighter anchoring of Lpp. Here, we show that Asp in position +2 and adjacent amino acids contribute in tightening the anchoring of Lpp by interaction of the negative charged Asp with the positive charged Lys‐PG.

## 1 INTRODUCTION

Bacterial lipoproteins (Lpp) belong to the class of lipid‐anchored proteins that in gram‐negative bacteria are attached in both the cytoplasmic and outer membranes, whereas in gram‐positive bacteria they are attached only in the cytoplasmic membrane (Hantke & Braun, 1973; Nguyen & Götz, 2016). Most Lpp are translocated across the cytoplasmic membrane by the bacterial SecYEG apparatus (Sugai & Wu, 1992), but few by the twin‐arginine translocation (Tat) pathway (Lee, Tullman‐Ercek, & Georgiou, 2006). The lipoprotein signal peptides possess at their C‐terminal end a conserved lipobox [–Leu/Val/Ile‐3–Ser/Ala‐2–Ala/Gly‐1–Cys+1], which is recognized by the modification machinery (Hayashi & Wu, 1990). This machinery comprises three enzymes: the diacylglyceryl transferase (Lgt), the signal peptidase II (Lps) and finally N‐acyltransferase (Lnt) (Hayashi & Wu, 1990; Inouye, Wang, Sekizawa, Halegoua, & Inouye, 1977). Lgt transfers the sn‐1,2‐diacylglyceryl group from phosphatidylglycerol to the prolipoprotein (Sankaran & Wu, 1994), Lsp recognizes the diacylglyceryl modification and cleaves between the amino acid at position –1 and the lipid‐modified cysteine (+1) residue (Hussain, Ichihara, & Mizushima, 1982), and Lnt carries out the *N*‐acylation of the free N‐terminus of cysteine to form *N*‐acyl diacylglyceryl cysteine (Gan et al., 1995). After the modification, the Lpp of gram‐negative bacteria either stay at the inner membrane or are exported to the outer membrane through the Lol (localization of lipoprotein) system (Narita, Matsuyama, & Tokuda, 2004; Tokuda & Matsuyama, 2004). However, Lpp that contain aspartate at position +2 (Asp+2) next to cysteine (+1) are hardly recognized by the Lol machinery. Therefore, these Lpp remain anchored at the inner membrane (Narita & Tokuda, 2011; Okuda & Tokuda, 2011). Thus in *E. coli*, the amino acid at position +2 determines the localization of Lpp either in inner or outer membrane (Yamaguchi, Yu, & Inouye, 1988). By in silico prediction it was assumed that the residues following the +1 cysteine may contain the Lpp sorting information (Zuckert, 2014).

Unlike gram‐negative bacteria, gram‐positive bacteria lack the outer membrane and the corresponding Lol machinery. Therefore, the influence of Asp+2 on Lpp sorting has not been explored. Since Lpp are the major surface proteins in gram‐positive bacteria, they play a crucial role in nutrient transport and in virulence (Nguyen & Götz, 2016; Shahmirzadi, Nguyen, & Götz, 2016). In gram‐positive pathogens Lpp play a crucial role in infection (Nguyen & Götz, 2016; Takeuchi, Hoshino, & Akira, 2000), sepsis (Angus & van der Poll, 2013; Schmaler et al., 2009), inflammation (Skabytska et al., 2014), and immune modulation via TLR2‐MyD88 activation (Hashimoto et al., 2006; Schmaler et al., 2009; Takeuchi et al., 2000).

While diacylated Lpp are sensed by the TLR2/TLR6 heterodimer, triacylated Lpp are sensed by the TLR2/TLR1 heterodimer (Jin et al., 2007; Kang et al., 2009; Schenk, Belisle, & Modlin, 2009; Takeda, Takeuchi, & Akira, 2002). As the Lpp receptors TLR2, TLR1, and TLR6 recognize Lpp via their ectodomain (Jimenez‐Dalmaroni et al., 2015) it is assumed that soluble Lpp in the bacterial supernatant bind to their receptors. It means that certain Lpp are not permanently anchored in the outer leaflet of the cytoplasmic membrane but are released into the environment during growth. Indeed, it has been shown recently that *S. aureus* strains that produce the detergent‐like phenol‐soluble modulins (PSMs) (Cheung, Joo, Chatterjee, & Otto, 2014) release higher amounts of Lpp from the cytoplasmic membrane (Hanzelmann et al., 2016).

As the amount of released Lpp into the environment is correlated with the immune response we asked the question whether negatively charged amino acids (aa) next to the cysteine in position +1 strengthens the anchoring of Lpp at the membrane by ionic interaction with positive charged phospholipids. To strengthen the hypothesis that the negative charged Asp+2 interacts with a positive charged membrane phospholipid, we created a Δ*mpr*F mutant in USA300. MprF lysinylates phosphatidylglycerol to Lys‐PG, one of the dominant membrane phospholipids in *S. aureus* (Ernst et al., 2015; Peschel et al., 2001; Staubitz, Neumann, Schneider, Wiedemann, & Peschel, 2004). In the *mprF* mutant the positive lysyl group is absent and now we saw that in this mutant Lpl8^+2D^ is not more retained to the membrane than Lpl8^+2S.^ This result clearly indicates that the strong retention of Lpl8^+2D^ to the membrane is most likely due to its ionic interaction with the positive charged Lys‐PG.

Furthermore, we screened all known Lpp of *S. aureus* USA300 for the +1 to +3 amino acid of the mature Lpp and found that Lpp with glycine at position +2 (G^+2^) were the most abundant in the supernatant, whereas Lpp with an Asp at position +2 (D^+2^) were hardly found in the supernatant (Nguyen et al., 2015; Nguyen et al., 2016; Stoll, Dengjel, Nerz, & Götz, 2005).

## 2 MATERIALS AND METHODS

### 2.1 Bacterial strains and growth conditions

Bacterial strains and plasmids used in this study are listed in Table 1. *S. aureus* strains were grown aerobically in basic medium, BM (1% soy peptone, 0.5% yeast extract, 0.5% NaCl, 0.1% glucose, and 0.1% K_2_HPO_4_, pH 7.4) at 37°C. For strains containing the plasmid pCtuf, the media were supplemented with 10 μg/ml of chloramphenicol.

**Table 1:** Strains and plasmids used in this study

Strains and plasmids	Description	References
Strains		
* E. coli* DC10B	A DNA cytosine methyltransferase mutant in the high‐efficiency *Escherichia coli* cloning strain DH10B	(Monk, Shah, Xu, Tan, & Foster, 2012)
* S. aureus* RN4220	A mutant of strain 8325‐4 that accepts the foreign DNA	(Thomas & Archer, 1989)
* *SA113∆*spa::erm*	Deletion of *spa* gene with erythromycin marker	(Schlag et al., 2010)
* *USA300∆*lpl*	Markerless deletion of lpl operon	(Nguyen et al., 2015; Nguyen et al., 2016)
* *USA300∆*lpl*∆*spa::erm*	Markerless deletion of lpl and deletion of spa gene	This study
* *USA300∆*lpl*∆*spa::erm*∆*mprF*	Clean deletion of *mpr*F gene in USA300∆*lpl*∆*spa*::*erm*	This study
Plasmids		
* *pBASE	Shutte vector for markerless gene deletion	(Bae & Schneewind, 2006)
* *pBASE‐*mpr*F	Knock‐out plasmid for replacement of *mpr*F	This study
* *pCtuf	Constitutive expression vector for staphylococci	(Biswas et al., 2006)
* *pCtuf‐lpl8^+2D^strep	Constitutive expression of *lpl*8 wild type	This study
* *pCtuf‐lpl8^+2S^strep	Constitutive expression of *lpl*8 with serine at +2 position	This study
* *pCtuf‐SitC^+2D^strep	Constitutive expression of *sitC* with aspartate in +2 position	This study
* *pCtuf‐SitC^+2G^strep	Constitutive expression of *sitC* wild type	This study

### 2.2 Creation of a double mutant *S. aureus* USA300∆*lpl*∆*spa::erm* by phage transduction

In order to avoid unnecessary binding of IgG to protein A, the *spa* gene was deleted by phage transduction using Φ11 with SA113 *spa*::*erm* (Schlag et al., 2010) as donor strain; as a result strain USA300∆*lpl*∆*spa::erm* was created. Briefly, phage Ф11 was used to produce a phage lysate of SA113∆*spa*::*erm*. The lysate was filtered through a 0.2 μm pore‐size filter and used to infect strain USA300∆*lpl* at a low multiplicity of infection (phage‐to‐recipient ratio of 1:10). Transducants carrying ∆*spa*::*erm* were selected on tryptic soya agar (TSA) supplied with erythromycin 2.5 μg/ml. As a control, the phage lysate was plated alone to avoid reisolating the donor strain. Positive clones containing the *spa* deletion were confirmed by DNA sequencing. For that, genomic DNA was isolated from clones using Quick‐gDNA^™^ Miniprep Kit (Zymo Research Europe GmbH) and used as template for PCR, using the primers For.spa.seq (5’‐AAGACCATGCTGAACAATTATTAGCTCA‐3’) and Rev.spa.seq (5’‐TGCAGGTGGTGTAGCAGCGAAAC‐3’). The PCR products were purified using illustra GFX PCR DNA and Gel Band Purification Kits (GE healthcare) and send for sequencing (GATC Biotech AG) to confirm *spa* deletion. All primers were purchased from integrated DNA technologies (Idt, Illinois).

### 2.3 Deletion of *mpr*F gene by allelic replacement in *S. aureus* USA300∆*lpl*∆*spa*::*erm*

The deletion of *mpr*F gene in *S. aureus* USA300∆*lpl*∆*spa*::*erm* was generated by homologous recombination. First, pBASE‐*mpr*F knockout plasmid containing the upstream and downstream flanking region was constructed. The 1000 bp upstream fraction was amplified by PCR using primer pairs F_up (5’‐GGAATT CCGGAGCTCGGTACTCTACTTGAAAAAATGAGTGTTC‐3’) and R_up (5’‐TTAATTATTTGTGCTGATTCATTTTTTCACATCAATTC‐3’) and the 741 bp downstream using F_down (5’‐AATGAATCAGCACAAATAATTAAAATCCAAGTGC‐3’) and R_down (5’‐CGACAGATCTGCGCGCTAGCTACTAAGGTCTAATGAAAGGATG‐3’). These two fragments were ligated into linearized pBASE by Gibson Assembly method. Briefly, the mixture of purified PCR flanking fragments and linearized plasmid were mixed 2xHiFi DNA Assembly Master Mix (New England Biolabs Inc., UK) at ratio 1:1 and incubated at 50°C for 1 hr. Later the ligation mixture was transformed into chemo‐competent *E. coli* DC10B and plated onto BM Ampicillin (100 μg/ml) plate and incubated at 37°C. The clones containing plasmid pBASE‐*mpr*F were screened using colony PCR and confirmed by DNA sequencing. The correct plasmid pBASE‐*mpr*F subsequently transformed by electroporation into USA300∆*lpl*∆*spa*::*erm*. The deletion procedure of *mpr*F gene was followed as described previously (Bae & Schneewind, 2006). The final gene deletion was checked and confirmed by PCR and DNA sequencing.

### 2.4 Construction of pCtuf‐Lpl8‐strep and pCtuf‐SitC‐strep

**Figure 1:**
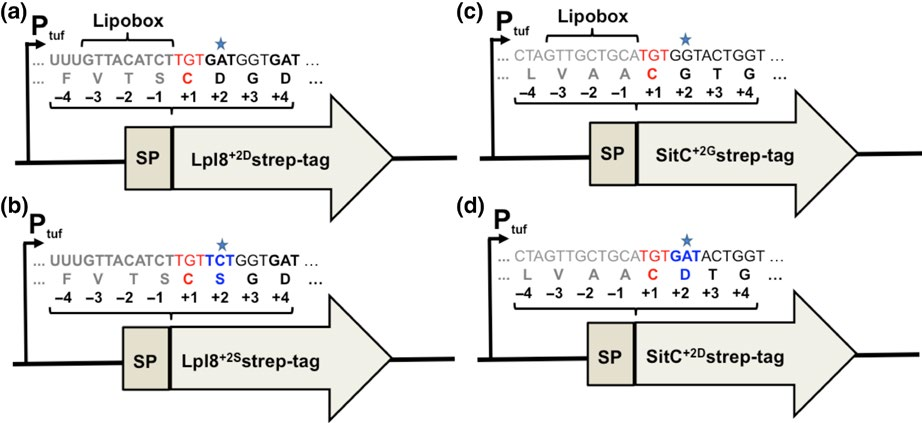
Schematic representation of plasmid constructs used in the study for lipoprotein release in *S. aureus*. The initial plasmid pCtuf were inserted with either (a) lpl8^+2D^, (b) lpl8^+2S^, (c) SitC^+2G^, and (d) SitC^+2D^, all with c‐terminal strep‐tag sequence (See methods for detail). The expression of Lpl8 and SitC variant were under the constitutive control of elongation tufA promoter

To construct the plasmid pCtuf for expression of Lpl8, the *lpl*8^+2D^ sequence was amplified from *S. aureus* USA300 genomic DNA, using the primers: Forward primer F_lpl8^+2D^ (5’‐AATATTTAATTAATGAAGTCTATAAAAAGGATTGGATTG‐3’) and Reverse primer R_lpl8‐strep (5’‐ ATTAAGCTTATTATT**TTTCAAATTGTGGATGTGACCATT**TATCCTCGCTTGGATTAAAG ‐ 3’) (restriction sites PacI and HindIII are underlined, respectively). The strep‐tag nucleotide sequence (bold letters) was added in the reverse primer for downstream protein detection. To develop the Lpl8 containing Serine at +2 position (Ser+2), the serine at the 24th codon was replaced into aspartate (D24S) using the forward primer F_lpl8^+2S^ (5’‐ataTTTAATTAATGAAGTCTATAAAAAGGATTGGATTGTGCATTAGTTTGTTGATTTTAATCATCTTTGTTACATCTTGT**TCT**GGTGATAATAAG‐3’) with the substituted nucleotide sequence (bold letters) and restriction site PacI in underline. The *lpl*8^+2S^ sequence was amplified with a pair of primers F_lpl8^+2S^ and R_lpl8‐strep. The amplified fragments and plasmid pCtuf were cut using the same digestion enzymes (PacI and HindIII) and subsequently ligated together (T4 Rapid ligation Kit by Thermo Scientific). The ligation products were transformed into *S. aureus* RN4220 by electroporation to yield the plasmid pCtuf‐*lpl*8^+2D^ and pCtuf‐*lpl*8^+2S^, which were then transformed into *S. aureus* USA300Δ*lpl* **∆** *spa::erm* (Figure 1a and b). For PCR amplification of *sitC* ^+2G^ sequence, forward primer F_SitC^+2G^ (5’‐AATATTAATTAATGAAAAAATTAGTACCTTTATTATTAGCCT‐3’) and reverse primer R_SitC‐strep (5’‐AATTAAGCTTATTATT**TTTCAAATTGTGGATGTGACCATT**TCATGCTTCCGTGTACAGTTTCAATATTT‐3’) were used. Restriction sites PacI and HindIII are underlined, respectively, and in the reversed primer strep‐tag nucleotide sequence was added (bold letters). To create *Sit*C sequence with Asp+2, the glycine at the 19th codon was replaced by the aspartate codon (G19D) using the forward primer F_SitC^+2D^ (5’‐ATATTAATTAATGAAAAAATTAGTACCTTTATTATTAGCCTTATTACTTCTAGTTGCTGCATGT**GAT**ACTG‐3’) with the substituted nucleotide sequence (bold letters) and restriction site PacI underlined. The *sitC* ^+2D^ sequence was amplified with a pair of primers F_SitC^+2D^ and R_SitC‐strep. Two resulting plasmid pCtuf‐SitC^+2G^ and pCtuf‐SitC^+2D^ were cloned into *S. aureus* RN4220 and later in USA300Δ*lpl* **∆** *spa::erm* (Figure 1c and d). The strain carrying the pCtuf plasmid without inserted fragment was used as negative control.

### 2.5 Harvesting of extracellular proteins

For the detection of extracellular proteins *S. aureus* clones were grown aerobically in BM with 10 μg/ml chloramphenicol at 37°C. Overnight cultures were diluted 1:100 in 100 ml BM broth and incubated aerobically at 37°C. Samples were taken at 4, 8, and 16 hr and OD_578 nm_ was determined at each time point. Samples were centrifuged at 5,000*g*/10 min at 4°C, filtered through 0.22 μm filters (Sarstedt, Nümbrecht). The amount of supernatant proteins was determined and adjusted to the same concentration. The filtered supernatants were kept at −80°C. The samples were thawed and prepared as described earlier (Nguyen et al., 2015).

### 2.6 Western blot

For Western blot analysis, the supernatants were thawed on ice and precipitated by 10 μl Strata clean resin (Agilent, Waldbronn). For SDS PAGE, the precipitated proteins were dissolved in 50 μl sample loading dye (3 X Laemmli beta‐mercaptoethanol), boiled for 7 min and stored on ice. 10 μl of each sample was loaded on 12% SDS‐PAGE. After gel electrophoresis proteins were transferred onto nitrocellulose membrane (Protran, GE, Frankfurt am Main) using Trans Blot device (Bio‐Rad, Munich) at 350 V for 40 min. Membranes were then blocked overnight with RotiBlock blocking reagent (Roth, Karlsruhe). Membranes were incubated with primary antibody (anti‐strep‐tag rabbit IgG, Abcam) for 1 hr and subsequently with secondary antibodies (anti‐rabbit IgG goat IgG alkaline phosphatase conjugated, Sigma) for 1 hr each at room temperature under gentle shaking. Detection was carried out using BCI/NBP (Sigma, Munich); blots were scanned by Epson scanner.

## 3 RESULTS

### 3.1 The lipoproteins with Asp at position +2 are not frequent in gram‐positive bacteria

First, we investigated how frequent is Asp+2 is gram‐positive Lpp. We used PRED‐LIPO prediction program for analyzing Lpp of gram‐positive bacteria (http://biophysics.biol.uoa.gr/PRED-LIPO-results/) (Bagos, Tsirigos, Liakopoulos, & Hamodrakas, 2008). The program includes Lpp data of 179 gram‐positive species and strains. There are three species representatives that lacked Lpp: The mycoplasma‐like plant pathogens *Aster yellows witches‐broom phytoplasma* AYWB and *Onion yellows phytoplasma* as well as the lactic acid bacterium *Oenococcus oeni* PSU‐1. The other of the 176 species/strains contain a wide variety of 9–160 Lpp. Only 62 out of 176 species/strains contain Lpp with Asp (+2). The number of Lpp with Asp+2 was very low ranging from 0% to 6% per species/strain (Table S1).

**Figure 2:**
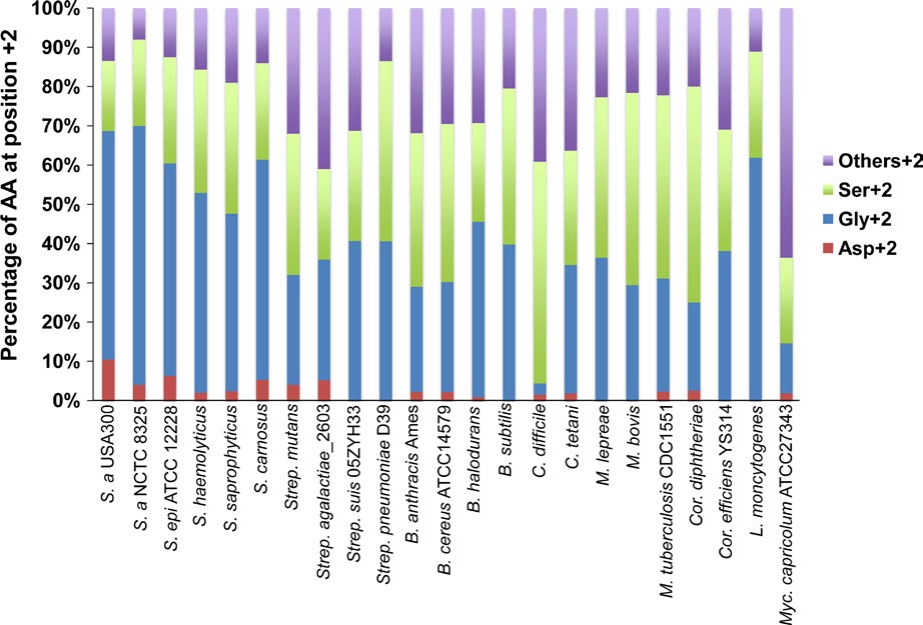
The dissemination of Lpp with aa at position +2 in gram‐positive species/strains. The percentages of Lpp with four groups of aa (Asp, Gly, Ser, and others) per total number of Lpp of several gram‐positive strains were shown. The calculation based on the PRED‐LIPO data. The detail data of Lpp with Asp+2 in 179 species/strains are presented in Table S1. S., Staphylococcus; Strep., Streptococcus; B., Bacillus; C., Clostridium; M., Mycobacterium; Cor., Corynebacterium; L., Listeria; Myc., Mycoplasma

To illustrate the low distribution of Lpp with Asp+2 we show a selection of gram‐positive species representative (Figure 2). As can be seen in the average the occurrence of Lpp with (Asp+2) ranged from 0% to 6%. Exceptionally high percentage (7 Lpp ≈ 10%) was the number of Lpp with (Asp+2) in the multiresistant and epidemic *S. aureus* USA300. Five of the seven Lpp with Asp+2 are encoded in the *lpl* gene cluster that is only found in the *S. aureus* species (Nguyen et al., 2015; Nguyen et al., 2016). The number of *lpl* genes in this cluster varies from 3 to 10 among the strains. Unlike in gram‐negative bacteria the number of Lpp with Asp+2 is limited in gram‐positive bacteria. Using *S. aureus* USA300 as a prototype of pathogenic *S. aureus* strains we found that among the 67 Lpp the most abundant amino acid at position +2 was glycine (39 Lpp), followed by serine (12 Lpp), aspartate (7 Lpp), alanine (3 Lpp), and each one with arginine, threonine, glutamine, asparagine, glutamate, and valine (Table 2).

**Table 2:** List of amino acids (aa) at the position +2 in *S. aureus* USA300 Lpp

aa at position +2^a^	Frequency	Number of detected Lpp in exoproteome^b^	References
G	39	29	(Becher et al., 2009; Burlak et al., 2007; Diep et al., 2014; Enany, Yoshida, & Yamamoto, 2014; Hanzelmann et al., 2016; Hempel, Herbst, Moche, Hecker, & Becher, 2011; Kusch & Engelmann, 2014; Le Marechal et al., 2011; Muthukrishnan et al., 2011; Pocsfalvi et al., 2008; Ravipaty & Reilly, 2010; Sibbald et al., 2006; Ziebandt et al., 2010)
S	12	9	
D	**7**	**0**	
A	3	3	
R	1	1	
T	1	1	
Q	1	0	
N	1	0	
E	1	0	
V	1	0	
Total	67	43	

aa, amino acids; G, glycine; S, serine; D, aspartate; A, alanine; R, arginine; T, threonine; Q, glutamine; N, asparagine; E, glutamate; V, valine.

a The frequency of amino acid at +2 position out of 67 predicted and approved Lpp of *S. aureus* USA300.

b The number of Lpp detected in the exoproteome of various *S. aureus* strains.

### 3.2 Evaluation of *S. aureus* Lpp detected in exoproteome

Next we analyzed the published exoproteomic data of *S. aureus* to see which Lpp with amino acid in position +2 are preferentially found in the supernatant. It turned out that in the supernatant particularly Lpp with uncharged or positively charged aa in position 2 were found: such as glycine, serine, alanine, arginine, and threonine. Lpp with aspartate in position 2 (Asp+2) were not detected in the exoproteome of various *S. aureus* strains. The results are summarized in Table 2, and the full detailed list of Lpp is shown in Table 3.

**Table 3:** Lpp detected in culture supernatant of *Staphylococcus aureus* strains either directly or via immunoproteomics

No	Locus tag in USA300	Function/ Annotation	aa sequence after Cysteine	Transmembrane domain (TMD)	Working strains	Exoproteome	References
1.	SAUSA300_1978	Ferric‐hydroxamate receptor/ FhuD1	**C** SASV	No TMD	D30 strain 930918‐3 USA300	Detected	(Hanzelmann et al., 2016; Muthukrishnan et al., 2011)
2.	SAUSA300_2235	Iron compound ABC transporter, iron compound‐binding protein FhuD2	**C** GNQG	No TMD	COL O46 O11 USA300 D30 strain 930918‐3 Multiple *S. aureus* strains	Detected	(Becher et al., 2009; Hanzelmann et al., 2016; Hempel et al., 2011; Kusch & Engelmann, 2014; Le Marechal et al., 2011; Muthukrishnan et al., 2011)
3.	SAUSA300_0721	Transferrin receptor/ SstD	**C**GNNS	No TMD	COL D30 USA300 Multiple *S. aureus* strains	Detected	(Hanzelmann et al., 2016; Hempel et al., 2011; Kusch & Engelmann, 2014; Muthukrishnan et al., 2011)
4.	SAUSA300_0117	Fe ABC transporter / SirA	**C** SGNS	No TMD	COL O46 O11 USA300	Detected	(Diep et al., 2014; Hanzelmann et al., 2016; Hempel et al., 2011; Le Marechal et al., 2011)
5.	SAUSA300_1032	Fe ABC transporter/ IsdE	**C** QSSS	No TMD	––––	Not‐detected	––––
6.	SAUSA300_0344	FepA, Fe‐binding protein, part of fepABC and tat‐AC cluster	**C** GNDD	No TMD	––––	Not‐detected	––––
7.	SAUSA300_2136	Fe ABC transporter	**C** GNTD	No TMD	COL MRSA clinical isolates USA300 O46 O11 Multiple *S. aureus* strains	Detected	(Becher et al., 2009; Diep et al., 2014; Hanzelmann et al., 2016; Hempel et al., 2011; Herbert et al., 2010; Kusch & Engelmann, 2014; Le Marechal et al., 2011)
8.	SAUSA300_0219 nq	Iron Binding Protein	**C** ANTR	No TMD	D30 strain 930918‐3	Detected	(Muthukrishnan et al., 2011)
9.	SAUSA300_0618	Manganese‐binding protein MntC (SitC)	**C** GTGG	No TMD	O46 O11 D30 strain 930918‐3 USA300	Detected	(Diep et al., 2014; Hanzelmann et al., 2016; Le Marechal et al., 2011; Muthukrishnan et al., 2011)
10.	SAUSA300_2351	Zinc‐binding, adcA‐like	**C** GNDD	No TMD	USA300	Detected	(Hanzelmann et al., 2016)
11.	SAUSA300_2411	Cobalt and nickel transporter Cnt (Opp1A)	**C** GGNK	No TMD	COL O46 O11 USA300	Detected	(Diep et al., 2014; Hanzelmann et al., 2016; Hempel et al., 2011; Le Marechal et al., 2011)
12.	SAUSA300_0231	Nickel ABC transporter	**C** GSMH	No TMD	D30 strain 930918‐3 USA300	Detected	(Hanzelmann et al., 2016; Muthukrishnan et al., 2011)
13.	SAUSA300_0203	Nickel‐Peptide/ transporter substrate‐binding protein	**C** GVPT	No TMD	USA300	Detected	(Hanzelmann et al., 2016)
14.	SAUSA300_2230	Molybdenum ABC transporter (ModA)	**C** SNSN	No TMD	D30 strain 930918‐3 USA300	Detected	(Hanzelmann et al., 2016; Muthukrishnan et al., 2011)
15.	SAUSA300_1283	Thioredoxine reductase, phosphate ABC transporter, phosphate‐binding protein PstS	**C** GGGN	No TMD	Various *S. aureus* strains	Detected	(Sibbald et al., 2006)
16.	SAUSA300_0145	Phosphonate ABC transporter	**C** GNSS	No TMD	D30 strain 930918‐3	Detected	(Muthukrishnan et al., 2011)
17.	SAUSA300_0175	Nitrate ABC transporter substrate‐binding protein	**C D**WQR	No TMD	–––	Not‐detected	–––
18.	SAUSA300_2391	Glycine betaine /carnitine/ choline ABC transporter (OpuCc)	**C** SLPG	No TMD	D30 strain 930918‐3	Detected	(Hanzelmann et al., 2016; Muthukrishnan et al., 2011)
19.	SAUSA300_2359	Amino acid ABC transporter	**C** GNNS	No TMD	COL SA strain D30 SA strain 930918‐3 USA300 Multiple *S. aureus* strains	Detected	(Becher et al., 2009; Hanzelmann et al., 2016; Kusch & Engelmann, 2014; Muthukrishnan et al., 2011; Ravipaty & Reilly, 2010)
20.	SAUSA300_0073	Peptide ABC transporter	**C** GNGQ	No TMD	–––	Not‐detected	–––
21.	SAUSA300_0891	Oligopeptide ABC transporter (Opp3A)	**C** ANDD	No TMD	COL D30 strain 930918‐3 USA300 Multiple *S. aureus* strains	Detected	(Hempel et al., 2011; Kusch & Engelmann, 2014; Muthukrishnan et al., 2011)
22.	SAUSA300_0892	Oligopeptide ABC transporter (Opp4A)	**C** GKSS	No TMD	USA300	Not‐detected	–––
23.	SAUSA300_0437	NLPA/ D‐Methionine binding (GmpC)	**C** GGNN	No TMD	COL USA300	Detected	(Becher et al., 2009; Hanzelmann et al., 2016)
24.	SAUSA300_0798	D‐Methionine ABC transporter	**C** GNGN	No TMD	MSSA MRSA USA300	Detected	(Diep et al., 2014; Enany et al., 2014; Hanzelmann et al., 2016)
25.	SAUSA300_0209	Maltose ABC transporter	**C** GPNR	No TMD	–––	Not‐detected	––––
26.	SAUSA300_1884	CamS sex pheromone biosynthesis	**C** GNHK	No TMD	COL USA300	Detected	(Becher et al., 2009; Hanzelmann et al., 2016)
27.	SAUSA300_0963	Quinol oxidase, subunit II (QoxA)	**C** SNIE	2 TMD	USA300 Multiple *S. aureus* strains	Detected	(Hanzelmann et al., 2016; Kusch & Engelmann, 2014)
28.	SAUSA300_0693	Electron transfer domain/ SaeP	**C** GNSN	No TMD	Multiple strains COL MRSA clinical isolates O46 O11 USA300 Multiple *S. aureus* strains	Detected	(Pocsfalvi et al., 2008; Becher et al., 2009; Herbert, Ziebandt et al., 2010; Le Marechal et al., 2011; Diep et al., 2014; Hanzelmann et al., 2016)
29.	SAUSA300_1790	Foldase protein PrsA	**C** GASA	No TMD	COL O46 O11 USA300	Detected	(Becher et al., 2009; Diep et al., 2014; Hanzelmann et al., 2016; Le Marechal et al., 2011; Ravipaty & Reilly, 2010)
30.	SAUSA300_2354	Thioredoxin/ Protein disulfide‐isomerase	**C** GKKE	No TMD	USA300	Detected	(Hanzelmann et al., 2016)
31.	SAUSA300_2046	YidC (OxaA) ‐ essential protein	**C D**YSK	5 TMD	––––	Not‐detected	––––
32.	SAUSA300_1436	PhiSLT ORF144‐like	**C** GEKE	No TMD	USA300	Detected	(Hanzelmann et al., 2016)
33.	pUSA300_HOUMR0011	Membrane bound penicillinase BlaZ	**C** NSNS	No TMD	––––	Not‐detected	––––
34.	SAUSA300_0410	Lpl‐1 νSaα specific	**C** GKGN	No TMD	––––	Not‐detected	–––
35.	SAUSA300_0411	Lpl‐2 νSaα specific	**C D**SSS	No TMD	––––	Not‐detected	–––
36.	SAUSA300_0413	Lpl‐3 νSaα specific	**C D**KSS	No TMD	––––	Not‐detected	–––
37.	SAUSA300_0414	Lpl‐4 νSaα specific	**C D**GDN	No TMD	––––	Not‐detected	–––
38.	SAUSA300_0415	Lpl‐5 νSaα specific	**C D**SQS	No TMD	––––	Not‐detected	––––
39.	SAUSA300_0416	Lpl‐6 νSaα specific	**C E**SNK	No TMD	––––	Not‐detected	––––
40.	SAUSA300_0417	Lpl‐7 νSaα specific	**C** RNMK	No TMD	––––	Not‐detected	––––
41.	SAUSA300_0418	Lpl‐8 νSaα specific	**C D**GDN	No TMD	––––	Not‐detected	––––
42.	SAUSA300_0419	Lpl‐9 νSaα specific	**C** GNMK	No TMD	COL D30 USA300 Multiple *S. aureus* strains	Detected	(Becher et al., 2009; Hanzelmann et al., 2016; Kusch & Engelmann, 2014; Muthukrishnan et al., 2011; Ravipaty & Reilly, 2010)
43.	SAUSA300_2429	Tandem lpp	**C** VIMT	No TMD	–––	Not‐detected	––––
44.	SAUSA300_2430	Tandem lpp	**C** GMKK	No TMD	–––	Not‐detected	––––
45.	SAUSA300_0100	Tandem lpp/ Conserved staphylococcal antigen 1A (Csa1A)	**C** GIGK	No TMD	Various *S. aureus* strains MRSA clinical isolates	Detected	(Sibbald et al., 2006; Herbert, Ziebandt et al., 2010)
46.	SAUSA300_0101	Tandem lpp	**C** GMGK	No TMD	––––	Not‐detected	––––
47.	SAUSA300_0102	Tandem lpp	**C** GIGK	No TMD	––––	Not‐detected	––––
48.	SAUSA300_0103	Tandem lpp	**C** GIGK	No TMD	USA300	Detected	(Hanzelmann et al., 2016)
49.	SAUSA300_0079	Unknown function	**C** SNND	No TMD	USA300	Detected	(Hanzelmann et al., 2016)
50.	SAUSA300_0372	Unknown function	**C** GNDT	No TMD	D30 Multiple strains USA300 COL *S. aureus* COL MW2 and LAC	Detected	(Becher et al., 2009; Burlak et al., 2007; Hanzelmann et al., 2016; Hempel et al., 2011; Muthukrishnan et al., 2011; Pocsfalvi et al., 2008; Ravipaty & Reilly, 2010; Sibbald et al., 2006)
51.	SAUSA300_0377	Unknown function	**C** ASDQ	No TMD	USA300	Detected	(Hanzelmann et al., 2016)
52.	SAUSA300_1492	Unknown function	**C** GSQN	No TMD	USA300	Detected	(Hanzelmann et al., 2016)
53.	SAUSA300_0992	Cell‐wall binding lipoprotein	**C** TTDK	No TMD	COL D30 strain 930918‐3 USA300	Detected	(Becher et al., 2009; Hanzelmann et al., 2016; Muthukrishnan et al., 2011)
54.	SAUSA300_2403	Unknown function	**C** GKEQ	No TMD	USA300	Detected	(Hanzelmann et al., 2016)
55.	SAUSA300_0724	Unknown function	**C** GNKE	No TMD	MRSA clinical isolates	Detected	(Sibbald et al., 2006; Herbert, Ziebandt et al., 2010)
56.	SAUSA300_2315	Unknown function	**C** GQDS	No TMD	Multiple *S. aureus* strains USA300	Detected	(Hanzelmann et al., 2016; Kusch & Engelmann, 2014)
57.	SAUSA300_2614	Unknown function	**C** SKQN	No TMD	––––	Not‐detected	––––
58.	SAUSA300_0663	Unknown function	**C** GKSQ	No TMD	––––	Not‐detected	––––
59.	SAUSA300_1106	Unknown function	**C** SFGG	No TMD	COL MRSA clinical isolates USA300 Multiple *S. aureus* strains	Detected	(Becher et al., 2009; Herbert, Ziebandt et al., 2010)
60.	SAUSA300_0303	Unknown function	**C** SNKG	No TMD	––––	Not‐detected	––––
61.	SAUSA300_1478	Unknown function	**C** GQKY	No TMD	USA300	Detected	(Hanzelmann et al., 2016)
62.	SAUSA300_1376	Unknown function	**C** STTN	No TMD	MRSA clinical isolates	Detected	(Herbert, Ziebandt et al., 2010)
63.	SAUSA300_1379	Unknown function	**C** STTN	No TMD	––––	Not‐detected	–––––
64.	SAUSA300_1440	Unknown function	**C** STME	No TMD	Various *S. aureus* strains	Detected	(Sibbald et al., 2006)
65.	SAUSA300_1742	Unknown function	**C** GANQ	No TMD	O46 O11	Detected	(Le Marechal et al., 2011)
66.	SAUSA300_1741	Unknown function	**C** GANQ	No TMD	————	Not‐detected	————
67.	SAUSA300_0769	Unknown function	**C** GHHQ	No TMD	COL USA300	Detected	(Becher et al., 2009; Hanzelmann et al., 2016)

### 3.3 The positive charged lysyl‐phosphatidylglycerol (Lys‐PG) enhances Lpl8 retention

**Figure 3:**
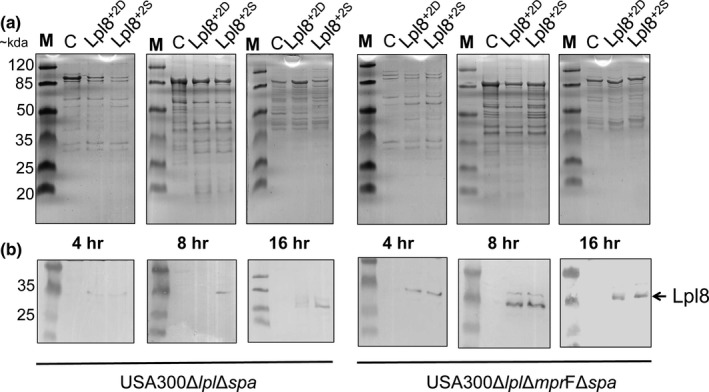
SDS‐PAGE profile of supernatant proteins from USA300 ∆*lpl*∆*spa*::*erm*. The extracellular proteins were harvested from the culture supernatant of USA300 ∆*lpl*∆*spa*::*erm* (left) and USA300 ∆*lpl*∆*spa*::*erm*∆*mpr*F (right) with pCtuf‐empty as control (C), pCtuf‐lpl8^+2D^strep (lpl8^+2D^) and pCtuf‐lpl8^+2S^strep (lpl8^+2S^) at three different time points and concentrated with Strata clean resin (Agilent, Waldbronn). Supernatant proteins were separated on SDS‐page: (A) Gels were stained with Coomassie blue stain and (B) The Western blot of anti‐streptag AB

In *S. aureus,* most of the Lpp containing Asp+2 belong to the Lpl lipoproteins. Previously it has been demonstrated that the Lpl lipoproteins are expressed both at transcriptional level and that they are associated with the cell envelope; their biological function is that they contribute to host cell invasion (Nguyen et al., 2015; Nguyen et al., 2016). To evaluate the effect of Asp+2 on Lpl release we substituted in Lpl8 (model Lpp) Asp+2 by Ser+2. Both genes encoding either Lpl8^+2D^ or the mutated Lpl8^+2S^ were provided with a Strep‐tag and were constitutively expressed on pCtuf vector in *S. aureus* USA300∆*lpl*∆*spa::erm* (Figure 1a and b). The release of Lpl8^+2D^ and Lpl8^+2S^ into the supernatant was monitored over time by Western blotting using Strep‐tag specific antibodies. For equal protein concentrations, the samples were adjusted to the same A280/260 values (Figure 3a). In the supernatants of the 4 hr cultures only faint bands were observed for Lpl8^+2D^ and Lpl8^+2S^. However, after 8 and 12 hr Lpl8^+2S^ was much more released than Lpl8^+2D^ (Figure 3b). The release of Lpl8^+2S^ occurred mainly in the late exponential and stationary growth phase.

We assume that the negatively charged Asp (+2) of Lpp interacts with the positive charged lysyl‐phosphatidylglycerol (Lys‐PG) that is synthesized by MprF in *S. aureus*. To prove this hypothesis, we created a Δ*mpr*F mutant in USA300∆*lpl*∆*spa::erm* and compared the release of Lpl8^+2D^ and Lpl8^+2S^ in USA300∆*lpl*∆*mpr*F∆*spa*. As expected, in the *mprF* mutant there is no difference in release of Lpl8^+2S^ the Lpl8^+2D^ to the supernatant at any time points tested (Figure 3b), indicating, that the positive charged Lysl‐PG increased the binding of Lpl8^+2D^ to the membrane.

### 3.4 The Asp (+2) has no effect on SitC release

**Figure 4:**
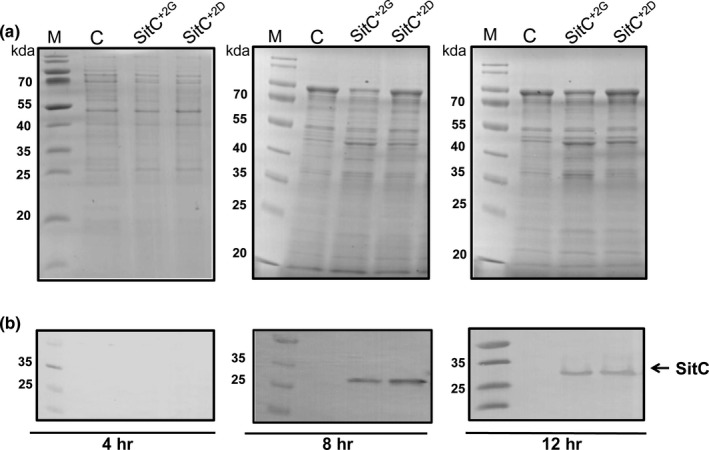
SDS‐PAGE profile of supernatant proteins from USA300 ∆*lpl*∆*spa*::*erm*. The extracellular proteins were harvested from the culture supernatant of USA300 ∆*lpl*∆*spa*::*erm* with pCtuf‐empty as control (C), pCtuf‐sitC^+2G^strep (sitC^+2G^) and pCtuf‐sitC^+2D^strep (sitC^+2D^) at three different time points (4, 8, and 16 hr) and concentrated with Strata clean resin (Agilent, Waldbronn). Supernatant proteins were separated on SDS‐page: (a) Gels were stained with Coomassie blue stain and (b) The Western blot of anti‐streptag AB

Next, we selected another Lpp candidate, SitC (MntC), one of the most abundant Lpp in *S. aureus* which is involved in manganese (Mn) transport (Horsburgh et al., 2002; Stoll et al., 2005). Two versions of SitC supplied with C‐terminal strep‐tag were constitutively expressed on pCtuf vector in *S. aureus* USA300∆*lpl*∆*spa::erm*: the original SitC with glycine at position +2 (SitC^+2G^) and the mutated SitC with aspartate at position +2 (SitC^+2D^) (Figure 1c and d). The release of SitC^+2G^ and SitC^+2D^ into the supernatant was monitored by Western blot in the same way as described for the Lpl8 variants. The protein samples were adjusted to the same A280/260 values to obtain an equal amount of extracellular proteins (Figure 4a). In 4‐hr culture supernatant, neither SitC^+2G^ nor SitC^+2D^ were detected in the Western blot, but after 8‐hr and 12‐hr cultivation SitC was clearly detected (Figure 4b). However, there was no difference observed in the release of both SitC variants.

## 4 DISCUSSION

Lipoproteins (Lpp) in gram‐positive bacteria have two major functions. They play an important role in physiology by being involved in transport of diverse nutrients, by acting as chaperons, by being involved in respiration, or by contributing to host cell invasion (Nguyen & Götz, 2016; Shahmirzadi et al., 2016). These functions are exerted by the protein part of the individual Lpp. Their second function is related to their interaction with the host's immune system. Here, it is not so much the protein part, but the lipid moiety plays the crucial role as potent activators of the innate and adaptive immune response by interacting with TLR2/1 or TLR2/6 receptors (Schenk et al., 2009; Takeda et al., 2002). Some Lpp were also considered as vaccine candidates. For example, immunization with FhuD2, a Lpp involved in ferric‐hydroxamate uptake, alone or together with hydroxamate siderophores, were protective in a murine staphylococcal infection model (Mishra et al., 2012). Recently, the combination of five antigens provided close to 100% protection against four different *S. aureus* strains (Bagnoli et al., 2015). Among them, were two Lpp, FhuD2 and the conserved staphylococcal antigen 1A (Csa1A).

Lpp in gram‐positive bacteria anchored in the outer leaflet of the cytoplasmic membrane. However, for the interaction with TLR2 receptor they must be released from the membrane to be able to expose the lipid moiety to the ectodomain of the TLR2‐(TLR1/6) heterodimers (Jimenez‐Dalmaroni et al., 2015; Jin et al., 2007). The less Lpp are released from the membrane the lower is the immune stimulation. We therefore asked the question how aa next to the invariable cysteine in position +1 contributes to the holding of Lpp to the membrane.

Here, we investigated the effect of aa at position +2 of the *S. aureus* Lpp in their release into the environment. In *E. coli* the significance of the aa at position +2 in withholding Lpp to the cytoplasmic membrane was well documented (Narita & Tokuda, 2016; Okuda & Tokuda, 2011). In several studies it has been reported that Asp+2 facilitates anchoring of Lpp at the inner membrane because Asp+2 functions as a Lol avoidance signal (Poquet, Kornacker, & Pugsley, 1993; Seydel, Gounon, & Pugsley, 1999; Terada, Kuroda, Matsuyama, & Tokuda, 2001; Yamaguchi et al., 1988). On the other hand, the outer membrane‐specific Lpp stimulated ATP hydrolysis by LolCDE but not the inner membrane‐specific Lpp (Masuda, Matsuyama, & Tokuda, 2002). The Lol avoidance mechanism was based on the strength of the hydrogen bonds between the negative charged Asp+2 and the positive charged phosphatidyl ethanolamine (PE) of the membrane phospholipids; glutamate at +2 position, with its longer side chain, interacts differently with PE (Hara, Matsuyama, & Tokuda, 2003). Therefore, the formation of a tight Lpp‐PE complex causes the Lol avoidance signal.

Like in *E. coli* there is in *S. aureus* also a predominant positive charged phospholipid, the Lys‐PG (Gould & Lennarz, 1970), which is synthesized by MprF (Ernst et al., 2015; Kuhn, Slavetinsky, & Peschel, 2015; Peschel et al., 2001). Lys‐PG, which yields 20%–40% of staphylococcus total membrane phospholipids, causes resistance against cationic antimicrobial compounds through ionic repulsion (Slavetinsky, Kuhn, & Peschel, 2016). Given that, gram‐positive bacteria lack the Lol system it is still possible that Asp+2 strengthens the anchoring of the corresponding Lpp to the membrane via the interaction with the positively charged Lys‐PG. Indeed, that is the case as in a *mprF* mutant there was no difference in retention between Asp+2 or Ser+2 in our model Lpl8.

Screening of Lpp of gram‐positive bacteria for Asp+2 revealed that this aa is very rare at this position, and, if at all, it is mainly found in pathogenic species/strains of *Bacillus*,* Clostridium*,* Mycobacterium*,* Staphylococcus aureus*, and some streptococci (Table S1). To verify the question, we screened 13 publications containing exoproteomic data of *S. aureus* and found out that none of seven Lpp with Asp+2 were detected in supernatant (Table 2 and 3). These seven Lpp with Asp+2 consist of; SAUSA300_0175 with a putative function as nitrate ABC transporter substrate‐binding protein, YidC (OxaI), and 5 Lpl lipoproteins. YidC, is an essential Lpp in bacteria acting as a membrane protein translocase and chaperone for membrane protein folding (Kuhn & Kiefer, 2017). Lpl lipoproteins contribute to *S. aureus* invasion to the host cells and G2/M transition delay (Nguyen et al., 2015; Nguyen et al., 2016). We assume that for the function of these seven Lpp with Asp+2, a tighter anchoring to the cytoplasmic membrane is necessary for their function. Particularly the epidemic *S. aureus* strains, such as USA300, which contain seven Lpp with Asp+2. Five of these Lpp are encoded in the *lpl* operon of the νSAα genomic island (Diep et al., 2006). This *lpl* operon contributes to host cell invasion via the protruding protein part (Nguyen et al., 2015; Nguyen et al., 2016). It makes sense that these Lpl proteins are especially tightly anchored to the cytoplasmic membrane to facilitate host cell invasion by interacting with the proposed target molecule at the host cell surface.

To experimentally verify the role of Asp+2 in retaining of Lpp at the cytoplasmic membrane we substituted the Lpl8^+2D^ by Lpl8^+2S^. We have chosen Ser as many Lpp with Gly or Ser in position +2 are found in the exoproteome (Table 2). However, when we substituted SitC^+2G^ by SitC^+2D^; there was no difference in release into the supernatant (Figure 4b). Apparently, aa in positions downstream of +2 might play a role. In Lpl8 for instance there is in position +4 a second aspartate **C**‐**D**G**D**N, whereas in SitC (MntC) **C**‐GTGG there is no Asp (Table 3). These results suggest that aspartate in position +2 plays a role but is not sufficient to withhold Lpp tightly at the cytoplasmic membrane and aa in position +3 and +4 might also contribute to this function.

In *E. coli* it has been shown that besides the +2, aa at position +3 also contributes in sorting of Lpp to the inner or outer membrane. Glu, Asp, Gln, or Asn at +3 position enhanced the retention to the inner membrane, whereas His, Lys, Val, Ile, Ala, Cys, or Thr decreased it (Terada et al., 2001). In *Pseudomonas aeruginosa* it was shown that Lys and Ser at positions +3 and +4 play a critical role for retaining Lpp in the inner membrane (Narita & Tokuda, 2007) (Lewenza, Mhlanga, & Pugsley, 2008). In *P. aeruginosa* Lpp that are located in the inner membrane have Gly+2 followed by Asp/Glu +3 (Remans, Vercammen, Bodilis, & Cornelis, 2010). Recently, it has been shown that aa variations in +2 of the alkaline phosphatase (PhoA) expressed in *Mycoplasma gallisepticum* did not affect the retention of PhoA to the membrane (Panicker, Kanci, Markham, & Browning, 2016). Finally, in *Bacillus subtilis* it was found that Gly+2 facilitates release of Lpp while a Ser+2 favors withholding in the membrane (Tjalsma & van Dijl, 2005).

## 5 CONCLUSION

**Figure 5:**
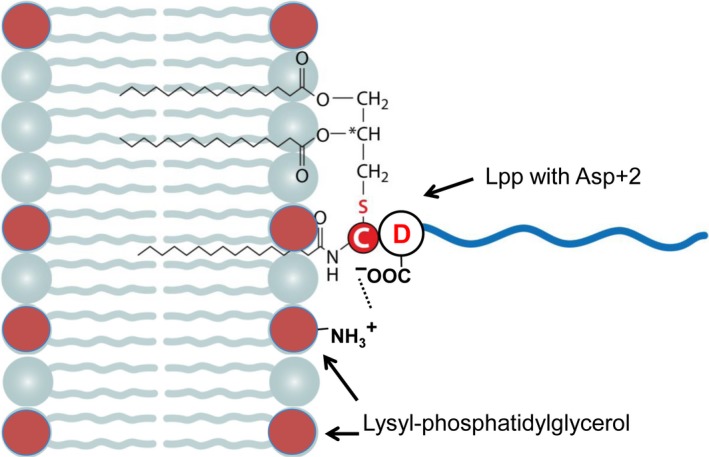
Model for the enforced interaction of Lpp Asp+2 (negative charged) with the Lys‐PG (positive charged). Phosphoglycerol (PG) in blue, Lys‐PG in red, D is Aspartate, C is Cysteine

An evaluation of literature data shows that in *S. aureus* the majority (75%) of Lpp found in the exoproteome carry Gly or Ser at position +2, whereas no Lpp with Asp in position +2 was found in the exoproteome. The role of Asp+2 in withholding Lpp to the cytoplasmic membrane was also confirmed by Lpl8^+2D^ and Lpl8^+2S^ in wild type but not in the *mprF* mutant. This suggests that the negative charged Asp withholds Lpp at the membrane by interacting with the positive charged Lys‐PG (Figure 5). On the other hand, substitution of SitC^+2G^ by SitC^+2D^ did not lead to a decreased release into the supernatant, suggesting that in staphylococci positions +3 to +5 might also be important for a more tightly anchoring of Lpp in the membrane. In gram‐positive bacteria the release of Lpp into the supernatant is crucial for the immune modulation via TLR2 activation thus contributing to inflammation and infection. On the other hand, tightly anchored Lpp such as Lpls’ or YidC is prerequisite for their function in host cell invasion and membrane protein sorting. Therefore, finding out sequence motives that modulate the strength of membrane anchoring is important.

## CONFLICT OF INTEREST

The authors declared that there is no conflict of interest.

## Supporting information

'
