# Supplementary material for: Aspartate tightens the anchoring of staphylococcal lipoproteins to the cytoplasmic membrane
Source: Microbiologyopen. 2017 Sep 13;6(6):e00525. doi: 10.1002/mbo3.525 (PMC5727369; doi:10.1002/mbo3.525)
Supplement: Supplementary file 1 — ' [file MBO3-6-na-s001.docx]

**Supplementary table S1**. Number of the Lpp with and without aspartate (D) at +2 position in Gram-positive bacteria according to the database of PRED-LIPO

|  | **Gram positive bacteria** | **No. of Lpp** | **No. of Lpp with +2 D** |
| --- | --- | --- | --- |
|  | *Acidothermus cellulolyticus* 11B | 27 | ---- |
|  | *Alkaliphilus metalliredigens* QYMF | 145 | ---- |
|  | *Arthrobacter aurescens* TC1 | 76 | ---- |
|  | *Arthrobacter* FB24 | 83 | ---- |
|  | *Aster yellows witches broom phytoplasma* AYWB | **0** | ---- |
|  | *Bacillus amyloliquefaciens* FZB42 | 67 | ---- |
|  | *Bacillus anthracis* Ames | 138 | 3 |
|  | *Bacillus anthracis* Ames 0581 | 138 | 3 |
|  | *Bacillus anthracis* str Sterne | 140 | 3 |
|  | *Bacillus cereus* ATCC14579 | 139 | 3 |
|  | *Bacillus cereus* ATCC 10987 | 139 | 3 |
|  | *Bacillus cereus* cytotoxis NVH 391 98 | 95 | 3 |
|  | *Bacillus cereus* E33L | 146 | 3 |
|  | *Bacillus clausii* KSM K16 | 141 | ---- |
|  | *Bacillus halodurans* | 123 | 1 |
|  | *Bacillus licheniformis* ATCC 14580 | 85 | ---- |
|  | *Bacillus licheniformis* DSM 13 | 87 | ---- |
|  | *Bacillus pumilus* SAFR 032 | 79 | ---- |
|  | *Bacillus subtilis* | 88 | ---- |
|  | *Bacillus thuringiensis* Al Hakam | 123 | 1 |
|  | *Bacillus thuringiensis* konkukian | 123 | 2 |
|  | *Bacillus weihenstephanensis* KBAB4 | 155 | 5 |
|  | *Bifidobacterium adolescentis* ATCC 15703 | 30 | ---- |
|  | *Bifidobacterium longum* | 33 | ---- |
|  | *Caldicellulosiruptor saccharolyticus* DSM 8903 | 19 | ---- |
|  | *Carboxydothermus hydrogenoformans* Z 2901 | 27 | ---- |
|  | *Clavibacter michiganensis* NCPPB 382 | 66 | ---- |
|  | *Clostridium acetobutylicum* | 74 | 2 |
|  | *Clostridium beijerinckii* NCIMB 8052 | 94 | 1 |
|  | *Clostridium botulinum* A | 71 | 3 |
|  | *Clostridium botulinum* AATCC 19397 | 65 | 2 |
|  | *Clostridium botulinum* A Hall | 63 | 2 |
|  | *Clostridium botulinum* F Langeland | 69 | 2 |
|  | *Clostridium difficile* 630 | 69 | 1 |
|  | *Clostridium kluyveri* DSM 555 | 49 | 1 |
|  | *Clostridium novyi* NT | 43 | ---- |
|  | *Clostridium perfringens* | 61 | 2 |
|  | *Clostridium perfringens* ATCC 13124 | 58 | 2 |
|  | *Clostridium perfringens* SM101 | 48 | 1 |
|  | *Clostridium phytofermentans* ISDg | 160 | ---- |
|  | *Clostridium tetani* E88 | 55 | 1 |
|  | *Clostridium thermocellum* ATCC 27405 | 69 | ---- |
|  | *Corynebacterium diphtheriae* | 40 | 1 |
|  | *Corynebacterium efficiens* YS-314 | 42 | ---- |
|  | *Corynebacterium glutamicum* ATCC 13032 Bielefeld | 90 | ---- |
|  | *Corynebacterium glutamicum* ATCC 13032 Kitasato | 89 | ---- |
|  | *Corynebacterium glutamicum* R | 85 | ---- |
|  | *Corynebacterium jeikeium* K411 | 47 | 2 |
|  | *Dehalococcoides* BAV1 | 20 | 1 |
|  | Dehalococcoides CBDB1 | 23 | 1 |
|  | *Dehalococcoides ethenogenes* 195 | 27 | ---- |
|  | *Deinococcus geothermalis* DSM 11300 | 42 | 1 |
|  | *Deinococcus radiodurans* | 42 | 1 |
|  | *Desulfitobacterium hafniense* Y51 | 153 | 1 |
|  | *Desulfotomaculum reducens* MI-1 | 52 | 1 |
|  | *Enterococcus faecalis* V583 | 76 | ---- |
|  | *Frankia alni* ACN14a | 48 | ---- |
|  | *Frankia* CcI3 | 39 | ---- |
|  | *Geobacillus kaustophilus* HTA426 | 65 | ---- |
|  | *Geobacillus thermodenitrificans* NG80-2 | 72 | 1 |
|  | *Kineococcus radiotolerans* SRS30216 | 57 | ---- |
|  | *Lactobacillus acidophilus* NCFM | 43 | ---- |
|  | *Lactobacillus brevis* ATCC 367 | 29 | ---- |
|  | *Lactobacillus casei* ATCC 334 | 47 | ---- |
|  | *Lactobacillus delbrueckii bulgaricus* | 25 | ---- |
|  | *Lactobacillus delbrueckii* bulgaricus ATCC BAA-365 | 26 | ---- |
|  | *Lactobacillus gasseri* ATCC 33323 | 30 | ---- |
|  | *Lactobacillus helveticus* DPC 4571 | 25 | ---- |
|  | *Lactobacillus johnsonii* NCC 533 | 40 | ---- |
|  | *Lactobacillus plantarum* | 49 | ---- |
|  | *Lactobacillus reuteri* F275 | 15 | ---- |
|  | *Lactobacillus sakei* 23K | 27 | 1 |
|  | *Lactobacillus salivarius* UCC118 | 20 | ---- |
|  | *Lactococcus lactis* | 33 | ---- |
|  | *Lactococcus lactis cremoris* MG1363 | 35 | ---- |
|  | *Lactococcus lactis cremoris* SK11 | 35 | ---- |
|  | *Leifsonia xyli* CTCB0 | 30 | ---- |
|  | *Leuconostoc mesenteroides* ATCC 8293 | 1 | ---- |
|  | *Listeria innocua* | 65 | ---- |
|  | *Listeria monocytogenes* | 63 | ---- |
|  | *Listeria monocytogenes* 4b F2365 | 58 | ---- |
|  | *Listeria welshimeri serovar* 6b SLCC5334 | 71 | ---- |
|  | *Mesoplasma florum* L1 | 21 | ---- |
|  | *Moorella thermoacetica* ATCC 39073 | 28 | ---- |
|  | *Mycobacterium avium* 104 | 67 | ---- |
|  | *Mycobacterium avium paratuberculosis* | 56 | ---- |
|  | *Mycobacterium bovis* | 51 | ---- |
|  | *Mycobacterium bovis* BCG Pasteur 1173P2 | 53 | ---- |
|  | *Mycobacterium gilvum* PYR-GCK | 95 | ---- |
|  | *Mycobacterium* JLS | 103 | ---- |
|  | *Mycobacterium* KMS | 101 | ---- |
|  | *Mycobacterium leprae* | 22 | ---- |
|  | *Mycobacterium* MCS | 95 | ---- |
|  | *Mycobacterium smegmatis* MC2 155 | 144 | 3 |
|  | *Mycobacterium tuberculosis* CDC1551 | 45 | 1 |
|  | *Mycobacterium tuberculosis* F11 | 50 | 1 |
|  | *Mycobacterium tuberculosis* H37Ra | 53 | 1 |
|  | *Mycobacterium tuberculosis* H37Rv | 53 | 1 |
|  | *Mycobacterium ulcerans* Agy99 | 59 | ---- |
|  | *Mycobacterium vanbaalenii* PYR | 111 | 1 |
|  | *Mycoplasma agalactiae* PG2 | 66 | 7 |
|  | *Mycoplasma capricolum* ATCC 27343 | 55 | 1 |
|  | *Mycoplasma gallisepticum* | 49 | ---- |
|  | *Mycoplasma genitalium* | 19 | ---- |
|  | *Mycoplasma hyopneumoniae* 232 | 27 | ---- |
|  | *Mycoplasma hyopneumoniae* 7448 | 22 | ---- |
|  | *Mycoplasma hyopneumoniae* J | 21 | ---- |
|  | *Mycoplasma mobile* 163K | 23 | ---- |
|  | *Mycoplasma mycoides* | 47 | ---- |
|  | *Mycoplasma penetrans* | 68 | ---- |
|  | *Mycoplasma pneumoniae* | 45 | ---- |
|  | *Mycoplasma pulmonis* | 46 | ---- |
|  | *Mycoplasma synoviae* 53 | 27 | ---- |
|  | *Nocardia farcinica* IFM10152 | 92 | ---- |
|  | *Nocardioides* JS614 | 96 | ---- |
|  | *Oceanobacillus iheyensis* | 125 | ---- |
|  | *Oenococcus oeni* PSU | **0** | ---- |
|  | *Onion yellows phytoplasma* | **0** | ---- |
|  | *Pediococcus pentosaceus* ATCC 25745 | 22 | ---- |
|  | *Pelotomaculum thermopropionicum* SI | 39 | ---- |
|  | *Propionibacterium acnes* KPA171202 | 49 | ---- |
|  | *Renibacterium salmoninarum* ATCC 33209 | 43 | ---- |
|  | *Rhodococcus* RHA1 | 142 | ---- |
|  | *Rubrobacter xylanophilus* DSM 9941 | 51 | ---- |
|  | *Saccharopolyspora erythraea* NRRL 2338 | 76 | ---- |
|  | *Salinispora tropica* CNB-440 | 61 | ---- |
|  | *Solibacter usitatus* Ellin6076 | 89 | ---- |
|  | *Staphylococcus aureus aureus* MRSA252 | 57 | 3 |
|  | *Staphylococcus aureus aureus* MSSA476 | 62 | 3 |
|  | *Staphylococcus aureus* COL | 61 | 3 |
|  | *Staphylococcus aureus* JH1 | 65 | 3 |
|  | *Staphylococcus aureus* JH9 | 65 | 3 |
|  | *Staphylococcus aureus* Mu50 | 66 | 3 |
|  | *Staphylococcus aureus* MW2 | 60 | 3 |
|  | *Staphylococcus aureus* N315 | 64 | 3 |
|  | *Staphylococcus aureus* NCTC 8325 | 50 | 2 |
|  | *Staphylococcus aureus* Newman | 64 | 7 |
|  | *Staphylococcus aureus* RF122 | 56 | 2 |
|  | *Staphylococcus aureus subsp. aureus* Mu3 | 66 | 3 |
|  | *Staphylococcus aureus* USA300 | 67 | 7 |
|  | *Staphylococcus carnosus* TM300 | 57 | 3 |
|  | *Staphylococcus epidermidis* ATCC 12228 | 48 | 3 |
|  | *Staphylococcus epidermidis* RP62A | 53 | 2 |
|  | *Staphylococcus haemolyticus* | 51 | 1 |
|  | *Staphylococcus saprophyticus* | 42 | 1 |
|  | *Streptococcus agalactiae* 2603 | 39 | 2 |
|  | *Streptococcus agalactiae* A909 | 34 | 1 |
|  | *Streptococcus agalactiae* NEM316 | 36 | 1 |
|  | *Streptococcus gordonii str. Challis substr*. CH1 | 58 | ---- |
|  | *Streptococcus mutans* | 25 | 1 |
|  | *Streptococcus pneumoniae* D39 | 37 | ---- |
|  | *Streptococcus pneumoniae* R6 | 35 | ---- |
|  | *Streptococcus pneumoniae* TIGR4 | 34 | ---- |
|  | *Streptococcus pyogenes* M1 GAS | 31 | ---- |
|  | *Streptococcus pyogenes* Manfredo | 31 | ---- |
|  | *Streptococcus pyogenes* MGAS10270 | 34 | ---- |
|  | *Streptococcus pyogenes* MGAS10394 | 30 | ---- |
|  | *Streptococcus pyogenes* MGAS10750 | 32 | ---- |
|  | *Streptococcus pyogenes* MGAS2096 | 30 | ---- |
|  | *Streptococcus pyogenes* MGAS315 | 31 | ---- |
|  | *Streptococcus pyogenes* MGAS5005 | 32 | ---- |
|  | *Streptococcus pyogenes* MGAS6180 | 34 | ---- |
|  | *Streptococcus pyogenes* MGAS8232 | 30 | ---- |
|  | *Streptococcus pyogenes* MGAS9429 | 31 | ---- |
|  | *Streptococcus pyogenes* SSI-1 | 26 | ---- |
|  | *Streptococcus sanguinis* SK36 | 55 | ---- |
|  | *Streptococcus suis* 05ZYH33 | 32 | ---- |
|  | *Streptococcus suis* 98HAH33 | 29 | ---- |
|  | *Streptococcus thermophilus* CNRZ1066 | 25 | ---- |
|  | *Streptococcus thermophilus* LMD-9 | 29 | ---- |
|  | *Streptococcus thermophilus* LMG 18311 | 28 | ---- |
|  | *Streptomyces avermitilis* | 142 | ---- |
|  | *Streptomyces coelicolor* | 160 | ---- |
|  | *Symbiobacterium thermophilum* IAM14863 | 56 | ---- |
|  | *Syntrophomonas wolfei* Goettingen | 41 | ---- |
|  | *Thermoanaerobacter tengcongensis* | 44 | ---- |
|  | *Thermobifida fusca* YX | 53 | ---- |
|  | *Tropheryma whipplei* TW08 | 9 | ---- |
|  | *Tropheryma whipplei* Twist | 9 | ---- |
|  | *Ureaplasma urealyticum* | 43 | ---- |

Predlipo website shows Lpp data of **179** Gram-positive species/strains. **3** species revealed no Lpp at all, **114** species/strains Lpp have NO Aspartate at +2 position, and **63** species/strains Lpp contain aspartate (D) at +2 position. *S. carnosus* Lpp data is not listed in predlipo website (unpublished data provided by Dr. Ralf Rosenstein).
